# Cgrp Uptake into Perivascular Capsaicin-Sensitive Nerve Terminals

**DOI:** 10.1100/tsw.2001.440

**Published:** 2001-11-10

**Authors:** A. Sams-Nielsen, C. Orskov, I. Jansen-Olesen

**Affiliations:** ^1^ Department of Pharmacology, The Royal Danish School of Pharmacy, Copenhagen, Denmark; ^2^ Department of Medical Anatomy, The Panum Institute, , University of Copenhagen, Denmark

## Abstract

Specific mechanisms, providing reuptake of cathecholamine and amino acid neurotransmitters (e.g., serotonin and glutamate) into cells of the central nervous system are well known, whereas neuronal uptake of neuropeptides have not previously been reported.

